# Effects of a Brain-Computer Interface With Virtual Reality (VR) Neurofeedback: A Pilot Study in Chronic Stroke Patients

**DOI:** 10.3389/fnhum.2019.00210

**Published:** 2019-06-19

**Authors:** Athanasios Vourvopoulos, Octavio Marin Pardo, Stéphanie Lefebvre, Meghan Neureither, David Saldana, Esther Jahng, Sook-Lei Liew

**Affiliations:** ^1^Neural Plasticity and Neurorehabilitation Laboratory, Occupational Science and Occupational Therapy, University of Southern California, Los Angeles, CA, United States; ^2^Department of Neurology, Stevens Neuroimaging and Informatics Institute, University of Southern California, Los Angeles, CA, United States

**Keywords:** brain-computer interfaces, virtual reality, action observation, stroke, neurorehabilitation

## Abstract

Rehabilitation for stroke patients with severe motor impairments (e.g., inability to perform wrist or finger extension on the affected side) is burdensome and difficult because most current rehabilitation options require some volitional movement to retrain the affected side. However, although these patients participate in therapy requiring volitional movement, previous research has shown that they may receive modest benefits from action observation, virtual reality (VR), and brain-computer interfaces (BCIs). These approaches have shown some success in strengthening key motor pathways thought to support motor recovery after stroke, in the absence of volitional movement. The purpose of this study was to combine the principles of VR and BCI in a platform called REINVENT and assess its effects on four chronic stroke patients across different levels of motor impairment. REINVENT acquires post-stroke EEG signals that indicate an attempt to move and drives the movement of a virtual avatar arm, allowing patient-driven action observation neurofeedback in VR. In addition, synchronous electromyography (EMG) data were also captured to monitor overt muscle activity. Here we tested four chronic stroke survivors and show that this EEG-based BCI can be safely used over repeated sessions by stroke survivors across a wide range of motor disabilities. Finally, individual results suggest that patients with more severe motor impairments may benefit the most from EEG-based neurofeedback, while patients with more mild impairments may benefit more from EMG-based feedback, harnessing existing sensorimotor pathways. We note that although this work is promising, due to the small sample size, these results are preliminary. Future research is needed to confirm these findings in a larger and more diverse population.

## Introduction

Stroke is a leading cause of adult long-term disability worldwide (Mozaffarian et al., 2015), and an increasing number of stroke survivors suffer from severe cognitive and motor impairments each year. This results in a loss of independence in their daily life, such as decreased ability to perform self-care tasks and decreased participation in social activities (Miller et al., 2010). Rehabilitation following stroke focuses on maximizing restoration of lost motor and cognitive functions and on relearning skills to better perform activities of daily living (ADLs). There is increasing evidence that the brain remains plastic at later stages after stroke, suggesting additional recovery remains possible (Page et al., 2004; Butler and Page, 2006). To maximize brain plasticity, several rehabilitation strategies have been exploited, including the use of intensive rehabilitation (Wittenberg et al., 2016), repetitive motor training (Thomas et al., 2017), mirror therapy (Pérez-Cruzado et al., 2017), motor-imagery (Kho et al., 2014), and action observation (Celnik et al., 2008), amongst others.

Recently, growing evidence of the positive impact of virtual reality (VR) techniques on recovery following stroke has accumulated (Bermúdez i Badia et al., 2016). When combined with conventional therapy, VR is able to effectively incorporate rehabilitation strategies such as intensity, frequency, and duration of therapy in a novel and low-cost approach in the stroke population (Laver et al., 2017). However, patients with low levels of motor control cannot benefit from current VR tools due to their low volitional motor control, range of motion, pain, and fatigue. Rehabilitation for these individuals is challenging because most current training options require some volitional movement to train the affected side, however, research has shown that individuals with severe stroke may receive modest benefits from action observation and brain-computer interfaces (BCIs) (Silvoni et al., 2011).

Merging BCIs with VR allows for a wide range of experiences in which patients can feel immersed in various aspects of their environment. This allows patients to control their experiences in VR using only brain activity, either directly (e.g., movement in VR through explicit control) or indirectly (e.g., modulating task difficulty level based on workload as implicit control) (Vourvopoulos et al., 2016; Friedman, 2017). This direct brain-to-VR communication can induce a sensorimotor contingency between the patient’s internal intentions and the environment’s responsive actions, increasing the patient’s sense of embodiment of their virtual avatar (Slater, 2009; Ramos-Murguialday et al., 2013).

After a stroke resulting in severe motor impairments (e.g., inability to perform wrist or finger extension on the affected side), research shows that action observation combined with physical training enhances the effects of motor training (Celnik et al., 2008), eliciting motor-related brain activity in the lesioned hemisphere, leading to modest motor improvements (Ertelt et al., 2007; Garrison et al., 2013). Moreover, action observation in a head-mounted VR increases motor activity in both healthy and the post-stroke brains (Ballester et al., 2015; Vourvopoulos and Bermúdez i Badia, 2016a).

In addition, neurofeedback through BCIs has been proposed for individuals with severe stroke because BCIs do not require active motor control. Research on BCIs for rehabilitation has shown that motor-related brain signals are reinforced by rewarding feedback so they can be used to strengthen key motor pathways that are thought to support motor recovery after stroke (Wolpaw, 2012). Such feedback has previously shown modest success in motor rehabilitation for severe stroke patients (Soekadar et al., 2015).

The most common brain signal acquisition technology used with BCIs in stroke patients is non-invasive electroencephalography (EEG) (Wolpaw, 2012), which provide a cost-effective BCI platform (Vourvopoulos and Bermúdez i Badia, 2016b). In BCI paradigms for motor rehabilitation, EEG signals related to motor planning and execution are utilized. During a motor attempt, the temporal pattern of the Alpha rhythm (8–12 Hz) desynchronizes. The Alpha rhythm is also termed Rolandic mu or the sensorimotor rhythm (SMR) when it is localized over the sensorimotor cortices of the brain. Mu rhythms (8–12 Hz) are considered indirect indications of the action observation network (Kropotov, 2016) and reflect general sensorimotor activity. Mu rhythms are often detected with changes in the Beta rhythm (12–30 Hz) in the form of event-related desynchronization (ERD), in which a motor action is executed (Pfurtscheller and Lopes da Silva, 1999). These EEG rhythms, or motor-related EEG signatures, are primarily detected during task-based EEG (i.e., when the patient is actively moving or imagining movement) and used for neurofeedback.

Further, neurofeedback-induced changes in brain activity have also been linked to changes in brain activity at rest. That is, after training one’s brain activity using neurofeedback, the intrinsic, resting brain activity (i.e., EEG activity in the absence of a task) may also show changes. This resting brain activity can be used to assess more generalized brain changes, and baseline resting-state signatures may be used to predict recovery (Wu et al., 2015) or response to treatments (Zhou et al., 2018). When combined with neural injury information, resting EEG parameters can also help predict the efficacy of stroke therapy.

In this study, our goal was to combine the principles of virtual reality and BCIs to elicit optimal rehabilitation gains for stroke survivors. We hypothesized that merging BCIs with VR should induce illusions of movement and a strong feeling of embodiment within a virtual body via the action observation network, activating brain areas that overlap with those controlling actual movement, which is important for mobilizing neuroplastic changes (Dobkin, 2007). Using a VR-based BCI, those with severe stroke impairments can trigger voluntary movements of the virtual arm in a closed neurofeedback loop. This helps to increase the illusion of one’s own movements through the coordination between one’s intention and the observed first-person virtual action. Therefore, we developed a training platform called REINVENT, which uses post-stroke brain signals that indicate an attempt to move and then drives the movement of a virtual avatar arm, providing patient-driven action observation in head-mounted VR (Spicer et al., 2017). Our previous work using REINVENT with healthy individuals indeed showed that the combination of VR integrated into a BCI encouraged greater embodiment, and greater embodiment was related to greater neurofeedback performance (Anglin et al., 2019).

For this study, we recruited four chronic stroke survivors to undergo a longitudinal BCI-VR intervention using REINVENT to provide EEG-based neurofeedback with simultaneous EMG acquisition. We assessed intervention results using clinical measures, Transcranial Magnetic Stimulation (TMS) and Magnetic Resonance Imaging (MRI) and compared these measures before and after the intervention. The purpose of this study was twofold. First, we sought to determine whether REINVENT is feasible for stroke patients to use across repeated sessions and second, whether REINVENT might be able to strengthen motor-related brain signals in individuals with differing levels of motor impairment after stroke.

## Materials and Methods

### Population

In this pilot study, we recruited four chronic stroke survivors (1 female, 3 male) with subcortical stroke (mean age: 60 ± 5.8 years old). The inclusion criteria included: (1) chronic (>6 months) stroke, between 18 and 80 years of age, (2) motor impairment in the upper limb, and (3) brain lesion as demonstrated by brain imaging. The exclusion criteria included: the presence of (1) intracranial metal, (2) epilepsy, (3) pregnancy, (4) cognitive impairments or psychiatric disorders, and (5) being unable to understand the instructions. All individuals were right-handed prior to the stroke, had a normal or corrected-to-normal vision, and were safe for MRI. Medications for spasticity were not permitted during the study intervention. The experimental protocol was approved by the University of Southern California Health Sciences Campus Institutional Review Board (IRB), and written informed consent was obtained from all patients upon recruitment in accordance with the 1964 Declaration of Helsinki. Patient demographics and stroke characteristics are described in Table 1.

**Table 1:** Patients demographics.

Patient number	FMA-UE^a^	SIS^b^	Time since stroke (months)	Stroke lesion location	Lesion size (volume mm^3^)	Lesion overlap with the CST in the damaged hemisphere (%)	Affected side
1	13	45	112	SC	5237	22	Left
2	28	35	186	SC	2686	15	Right
3	37	10	77	SC	4389	33	Right
4	49	40	59	SC	172	0	Right
Mean	31.8	32.5	108.5				–
SD	13.1	13.5	48.6				–

*FMA, Fugl-Meyer Assessment; FMA-UE, Upper Extremity; and SIS, Stroke Impact Scale physical domain. ^a^FMA-UE was scored on a scale up to 66 points. ^b^SIS was scored on a scale of 0–100. SC, subcortical stroke.*

### REINVENT System

#### System Architecture

We implemented a software architecture that could be tailored for stroke patients with different motor capabilities and rehabilitation needs. This system incorporated interfaces with different degrees-of-freedom (DoF) for training patients with: (1) no active movement, using EEG in a direct brain-to-VR interfacing, (2) weak muscle activation, using EMG in a muscle-to-VR interfacing, and (3) substantial active movement, using hand tracking. The VR paradigm included avatar personalization with different gender hand models and different skin tones to increase embodiment and expand demographic inclusivity. Building upon our previous VR BCI training paradigm (Spicer et al., 2017), we created this new version with a distributed architecture, making it hardware independent, in an open and modular design. This updated version makes it possible to integrate as many new interfaces as needed to keep up with the rapid pace of technology development. In this version, the data acquisition and processing modules are also independent of the VR task, communicating bidirectionally over a network layer. We acquired the electrophysiological signals from the hardware using a set of “satellite” clients (EEG, EMG) and sending them to the processing module(s) and a logger via the Lab Streaming Layer (LSL) protocol. After signal processing, the extracted features (i.e., EEG bands, EMG flexion detection) were sent through the same protocol to VR. The VR training environment streamed back to the network the following items: task score, task events (e.g., trial start, pause, complete), and rotational information of the VR hand controllers in three-dimensions.

**FIGURE 1:**
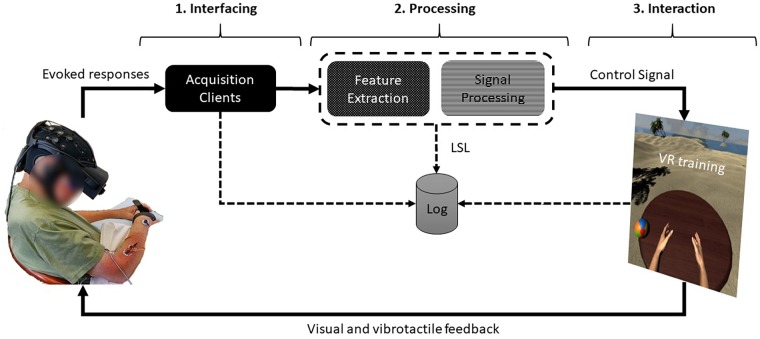
System architecture of a closed neurofeedback loop. From left, (1) the evoked physiological responses are captured at the interfacing layer through the data acquisition clients, (2) sent to the processing layer where the signals are filtered and logged, and then, (3) the extracted features (e.g., EEG bands) are sent to the interaction layer where VR training occurs. Written permission to use this photo was obtained from the individual.

All VR elements were implemented in the Unity game engine (Unity Technologies, San Francisco, CA, United States) and rendered through an Oculus Rift HMD using the Oculus SDK (Oculus VR, Menlo Park, CA, United States). Overall, the entire architecture layout was encapsulated in three inter-dependent layers: (1) interfacing, (2) processing, and (3) interaction. Each layer can incorporate a subset of independent subcomponents (e.g., device interfacing clients, custom processing code or out-of-the-box processing software, and desktop VR or mobile VR) (Figure 1).

#### Training Procedures and Tasks

We divided the experimental protocol is into three blocks: (a) pre-intervention, (b) intervention, (c) post-intervention.

**FIGURE 2:**
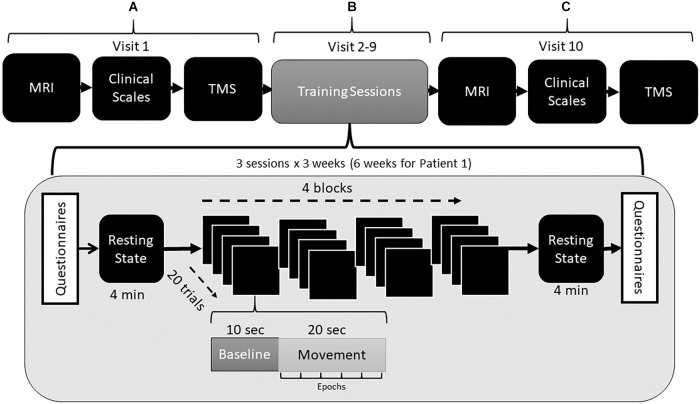
Experimental protocol in three levels. **(A)** Pre-intervention, including assessment through clinical scales, TMS, and MRI. **(B)** During each intervention session, questionnaires were completed to assess pre- and post-simulation sickness, followed by a resting-state session in which raw the EEG was recorded with the patients’ eye open and closed. Next, four training blocks consisting of 20 trials each were performed in VR. Resting-state and follow up questionnaires were then completed. **(C)** Post-intervention assessment with MRI and TMS.

In the pre- and post-intervention blocks, we assessed motor impairment in all the patients using a set of clinical tests. In addition, we acquired functional and structural scans during an MRI session and neurophysiological measurements during a TMS session (Figure 2A,C; see also sections Behavioral and Clinical Assessments and MRI and TMS Assessments).

In the intervention block, patients were trained over 8 sessions. Due to self-reported improvements in daily life activities, patient S01 requested a second intervention block of an additional 8 training sessions, leading in a total of 16 training sessions, lasting 6 weeks. However, for consistency across patients, only the first 8 sessions are reported in this study. A case report for this patient, including the results following the full 16 sessions, are presented separately (Vourvopoulos et al., 2019).

Each training session lasted for 1.5 h (Figure 2B). Before each session, patients were seated in a comfortable chair with a pillow under the affected arm for support. We instructed all patients to remain relaxed and avoid any unnecessary movement during the experiment. At the beginning and end of every training session, patients completed a set of questionnaires regarding simulator sickness. We also acquired a resting-state EEG acquisition (4 min).

The training task included a virtual environment in which a set of virtual hands represented the patient’s physical hands from a first-person perspective. During training, patients had to perform a wrist and elbow extension attempt of the virtual hand toward a pre-defined target, followed by rest. The patient’s virtual arm moved toward the target if their sensorimotor brain activity during the motor attempt increased relative to baseline. The intervention consisted of four blocks of training in VR (each block consisted of 20 trials), and each trial lasted 30 s. The first 10 s period was a resting baseline, during which the patient was asked to relax. We chose this duration because event-related changes need time to develop and then recover, and thus the inter-event interval between two consecutive motor events should last at least 10 s (Pfurtscheller and Lopes da Silva, 1999). The next 20 s consisted of a motor intervention, during which we asked the patient to try to move. This 20 s active movement period was divided into epochs of 4 s each, during which the average ERD was calculated and compared to the ERD of the preceding 10 s baseline. Again, a trial was successful and triggered positive neurofeedback of a virtual arm moving toward a target if the ERD during the active trial was greater than at baseline. In the current study, we collected both EEG and EMG signals, but only EEG was used to control the VR neurofeedback to assess the efficacy of the BCI paradigm across all patients.

The experimental setup consisted of a desktop computer (OS: Windows 10, CPU: Intel^®^ Core^TM^ i7-6700 at 4.00 GHz, RAM: 16GB DDR3 1600 MHz, Graphics: NVIDIA GeForce GTX 1080) running all the acquisition, processing, and VR software. An Oculus Rift HMD was used to deliver the VR feedback to the user. The HMD had two OLED displays, 1080 × 1200 resolution per eye, at 90 Hz refresh rate, and 110° field of view (the extent of the observable environment at any given time). The HMD also featured 6-DoF tracking (3-axis rotational tracking and 3-axis positional tracking) and integrated headphones with 3D spatial audio. Moreover, the Oculus Rift HMD also included two Oculus touch controllers with 6-DoF, delivering vibrotactile feedback to the users.

### Behavioral and Clinical Assessments

During pre- and post-intervention sessions, a battery of assessments targeting the patient’s upper extremity motor function, spasticity, stroke-related impacts on life and simulator sickness for VR were completed. All clinical assessments were performed by a trained occupational therapist. The clinical and behavioral assessments included the following:

• Fugl-Meyer Upper Extremity scale (FMA-UA). The FMA-UA is a scale (0–66) that evaluates motor impairment in post-stroke individuals. A higher score is reflective of less motor impairment (Fugl-Meyer et al., 1975).
Modified Ashworth Spasticity (MAS) scale. The MAS is a scale (0–4) that measures spasticity, or velocity-dependent movement, in patients with central nervous system lesions. A lower score indicates less spasticity in the assessed muscle group (Gregson et al., 1999). We measured the MAS in the wrist and elbow.
Stroke Impact Scale (SIS). The SIS is a 59-item instrument measuring the self-reported quality of life of stroke survivors across eight categories. In the current study, we only utilized the SIS-Physical domain. Each item is rated on a 5-point Likert scale, designed for repeated administration to track patient changes over time (Vellone et al., 2015). The results are reported on a scale of 0–100, with higher scores indicating the best self-reported quality of life.
The Simulator Sickness questionnaire (revised by the UQO Cyberpsychology Lab, 2013) includes 16 questions on a 0–3 Likert scale resulting in two sub-scales: Nausea (9 questions for a maximum of 27 points) and Oculo-Motor (7 questions for a maximum of 21 points) (Kennedy et al., 1993).
Finally, we qualitatively acquired the patient’s feedback regarding enjoyment and ease of use in a free-form comments section that patients completed after each session.

### Physiological Measurements

#### EEG Acquisition and Online Processing

We used the Starstim 8 (Neuroelectrics, Barcelona, Spain) system to capture EEG data. Starstim is a wireless hybrid EEG/tCDS 8-channel neurostimulator system with a triaxial accelerometer for the recording and visualization of 24-bit EEG data at 500 Hz. The spatial distribution of the electrodes followed the 10–20 system configuration (Klem et al., 1999), with electrodes placed over the frontal, somatosensory and motor areas: frontal-central (FC3, FC4), central (C3, C4, C5, C6), and central-parietal (CP3, CP4). The electrodes were referenced and grounded to the right ear lobe, and the electrode impedance was kept at less than 10 kΩ. Finally, the EEG system was connected via Bluetooth to the dedicated desktop computer for raw signal acquisition and processing.

The EEG signals were first acquired through the Neuroelectrics NIC-2 client before sending them to OpenVibe platform for online processing. We used a surface Laplacian for spatial filtering over the target location (C3 or C4) and the adjacent electrodes because the desynchronization of SMRs are enhanced and better localized with Laplacian (Pfurtscheller, 1988; McFarland et al., 1997). The online signal processing included a bandpass filter (8–24 Hz), which was then squared, averaged over 500 samples, and logarithmized. Finally, the output was sent via LSL to the VR client.

#### EEG-Based Neurofeedback Training Performance

We measured training performance based on the number of successful motor actions initiated by the virtual hand toward a target. As described previously, each virtual action was triggered when the ERD power exceeded the baseline measurement over C3 or C4 locations and was calculated with the following formula:

(1) $$\mathrm{Score}=\frac{1}{n}\left(\sum_{i=1}^{n}x_{i}\right)*100$$

where *n* the total number of trials and *x_i_* the successful motor actions.

#### EEG *Post hoc* Analysis

For the *post hoc* EEG analyses, we processed EEG signals in Matlab (The MathWorks, MA, United States) with the EEGLAB toolbox (Delorme and Makeig, 2004). After importing the data and channel information, we used a high-pass filter at 1 Hz to remove the “baseline drift,” followed by line-noise and harmonics removal at 60 Hz. To reject bad channels and correct continuous data, we used an artifact subspace reconstruction (ASR) method (Kothe and Jung, 2016). The missing channels were interpolated before re-referencing to average. Next, we performed an independent component analysis (ICA) to remove EOG, EMG, and ECG artifacts (Makeig et al., 1996).

To acquire the different EEG bands, we extracted the power spectral density (PSD) for the following frequency bands: alpha (8–12 Hz), beta (12–30 Hz), theta (4–7 Hz), and gamma (25–90 Hz).

In addition, we extracted the event-related synchronization/desynchronization (ERS/ERD) following the standard ERS/ERD method (Pfurtscheller and Aranibar, 1979) in the mu band between 8 and 12 Hz and the beta band between 12 and 30 Hz. Both mu and beta power were extracted over C3 and C4 electrode locations. ERD was calculated using the following formula:

(2) $$\mathrm{ERD}=({\mathrm{Power}}_{\mathrm{Motor Activity}}-{\mathrm{Power}}_{\mathrm{Baseline}})/{\mathrm{Power}}_{\mathrm{Baseline}}\times 100$$

With positive numbers for ERS and negative numbers indicating ERD.

Moreover, we extracted the event-related EEG data maps as a time/frequency representation of ERD/ERS between 8 and 24 Hz (Graimann et al., 2002). These maps are also known as ERSP (event-related spectral perturbation) and act as a generalization of the ERS/ERD (Makeig, 1993).

Furthermore, we assessed the hemispheric lateralization through a hemispheric asymmetry index (HA). HA was computed using both the relative EEG band power during rest and the ERD during motor task over C3 and C4 electrode locations. The power values were measured contralateral to the lesioned side and were subtracted from ipsilateral values of the non-affected side. For those with left hemiparesis, this index was calculated using the following formula:

(3) $${\mathrm{HA}}_{\mathrm{left}}=\mathrm{PowerC}4_{\mathrm{Left movement}}-\mathrm{PowerC}3_{\mathrm{Left movement}}$$

For those with right hemiparesis, the index was calculated using the following formula:

(4) $${\mathrm{HA}}_{\mathrm{right}}=\mathrm{PowerC}3_{\mathrm{Right movement}}-\mathrm{PowerC}4_{\mathrm{Right movement}}$$

Finally, we extracted HA for alpha during the resting-state because alpha oscillation at rest is associated with motor and cognitive performance in stroke patients (Dubovik et al., 2012, 2013; Mottaz et al., 2015).

#### EMG Acquisition and Online Processing

We acquired surface EMG at 2000 Hz using a Delsys Trigno Wireless System (Delsys, MA, United States) with their proprietary software. Each sensor incorporated differential Ag electrodes with amplification and filtering stages and a 16-bit A/D converter. Three-axes of acceleration data were also acquired with the same sensors at 150 Hz and 8-bit ADC resolution. Delsys Trigno EMG sensors were placed on the extensor digitorum comunis (EDC), flexor carpi ulnaris (FCU), biceps brachii (BB), and triceps brachii (TB) muscles of the paretic arm. Electrode positioning was selected by palpation while individually performing elbow and wrist flexion and extension and was confirmed by visual inspection of the EMG signals. Skin preparation involved shaving the selected area, cleaning with alcohol, and applying abrasive and conducting paste. Both processing pipelines were implemented with a custom-made script in Matlab (The MathWorks, MA, United States). All raw and processed data were streamed and recorded via LSL.

#### EMG *Post hoc* Analysis

Online processing was performed by reading 0.5 s of the EMG signal from EDC and BB, applying a DC-offset correction, performing full-wave rectification and comparing the mean of each epoch with a predefined threshold of muscle activation.

Offline processing of the EDC signal applied epoch extraction within the boundaries of the trial markers of “*baseline*” and “*active contraction*,” DC-offset correction, band-pass filtering within 10-500 Hz, and full-wave rectification. Finally, the mean absolute value (MAV) of the epoched EMG signal was extracted as the main feature (Veer and Sharma, 2016):

(5) $${\mathrm{EMG}}_{\mathrm{mean}}=\mathrm{MAV}=\frac{1}{N}\sum_{n=1}^{N}\left|x_{n}\right|$$

Where *x_n_* is the voltage read by the sensor at that point in time, for N samples.

### MRI and TMS Assessments

We also acquired additional neural assessments (MRI, TMS) during pre- and post-intervention sessions for each patient to assess any potential brain changes.

#### MRI Acquisition

Using MRI, we acquired an anatomical image (T1w MPRAGE), a T2-weighted anatomical MRI, a diffusion-weighted MRI, and a 7 min resting-state fMRI (rs-fMRI). MRI data were acquired on a 3T Prisma MRI scanner (Siemens, Germany) with a 32-channel head coil, using protocols from the Human Connectome Project (HCP)^1^. The MRI sequences acquired included the following: T1-weighted MPRAGE scan (208 sagittal slices, 0.8 mm thick, *TR* = 2400 ms, *TE* = 2.22 ms, flip angle = 8°, voxel size 0.8 × 0.8 × 0.8 mm); T2-weighted turbo spin-echo scan (208 sagittal slices, 0.8 mm thick, *TR* = 3200 ms, *TE* = 523 ms, flip angle = 8°, voxel size 0.8 × 0.8 × 0.8 mm); diffusion MRI scan (92 slices, 1.5 mm thick, *TR* = 3230 ms, *TE* = 89.20 ms, multi-band factor = 4, flip angle = 78°, voxel size 1.5 × 1.5 × 1.5 mm, with a gradient protocol with seven scans at *b* = 0 s/mm^2^, 47 at *b* = 1500 s/mm^2^, 46 at *b* = 3000 s/mm^2^, and a complete repetition with reversed phase encoding in the A-P direction); and a rs-fMRI scan (7 min and 6 s covering 520 volumes of 72 slices per scan; *TR* = 800 ms, *TE* = 37 ms, flip angle = 52°, voxel size 2 × 2 × 2 mm). For patients with a brain lesion in the left hemisphere, we flipped both structural and functional scans to the right hemisphere for the group analyses.

#### Lesion Map, Lesion Size, and Lesion Overlap

**FIGURE 3:**
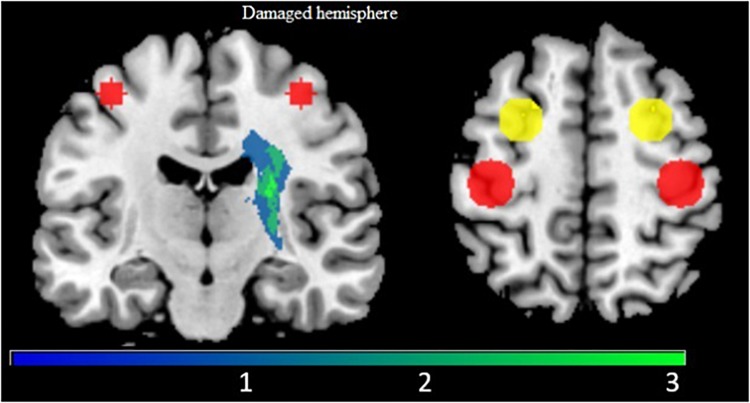
Lesion map does not overlap with cortical motor areas. Display of the lesion map (blue to green scale, with green indicating greater overlap across participants) with regions of interest for M1 (red) and PMd (Yellow). The M1 and PMd ROIs are used in ROI-to-ROI analysis (section fMRI ROI-to-ROI Analysis).

Using MRIcro software, we manually drew a lesion mask on each patient’s brain (see Figure 3). Then, using FSL we calculated the lesion size in mm^3^ (Table 1). Finally, we used the PALS toolbox (Ito et al., 2018) to calculate the overlap of each patient’s lesion with the corticospinal tract (CST), the primary descending motor pathway (Table 1). The lesion overlap with the CST has been shown to correspond to motor impairment after stroke (Riley et al., 2011).

#### Resting-State fMRI Processing

We analyzed all rs-fMRI data in Matlab using SPM12 and the CONN toolbox^2^. The preprocessing steps included slice-timing correction, motion realignment, noise correction using white matter, CSF and motion parameters as regressors, and band-pass filtering (0.01–0.1 Hz). We also performed co-registration between the functional scans and the 3D-T1w MPRAGE scans of each patient. Finally, we normalized the functional scans to MNI space and smoothed them using a Gaussian filter of 6 mm. To measure functional changes in the motor network, we compared the FC between regions of interest (ROI-to-ROI analyses).

#### ROI-to-ROI Analyses

We used four regions of interest (ROIs): left M1, right M1, left PMd, and right PMd. We used a meta-analysis from Hardwick and collaborators to define the location of the ROIs in the left hemisphere and then flipped them to the right hemisphere. The exact coordinates of the center of each ROI, which was defined as a sphere with a radius of 10 mm, were: left M1 (*x* = −38, *y* = −24, *z* = 56), right M1 (*x* = 38, *y* = −24, *z* = 56), left PMd (*x* = −26, *y* = 2, *z* = 60), right PMd (*x* = 26, *y* = 2, *z* = 60). In addition, to explore changes in the intra and inter-hemispheric connectivity in the motor network pre- and post-intervention sessions, we also classified these ROI-to-ROI interactions as follows: (1) damaged intra-hemispheric motor network connectivity: damaged M1-damaged PMd; (2) undamaged intra-hemispheric motor network connectivity: undamaged M1-undamaged PMd; and (3) inter-hemispheric motor network connectivity: average of (undamaged M1-damaged PMd connectivity, damaged M1-undamaged PMd connectivity, undamaged M1-damaged M1 connectivity, undamaged PMd-damaged PMd connectivity).

#### Transcranial Magnetic Stimulation (TMS)

We used single- and paired-pulse TMS (Magstim 200^2^ device with BiStim module; Magstim Inc., United Kingdom) with a figure-of-eight coil combined with the Brainsight neuronavigation system (Rogue Resolutions Ltd., United Kingdom) and surface electrodes to record muscle activity (EMG; Delsys Trigno wireless sensors, Delsys Inc., MA, United States). To localize the motor hotspot, we recorded EMG at the right first dorsal interosseous (FDI). We then recorded the patient’s resting motor threshold (RMT) and recruitment curve in each hemisphere by acquiring 5 MEPs at each of the following threshold 100, 110, 120, 130, 140, and 150% of the RMT.

### Diffusion MRI

In addition, we acquired diffusion MRI in order to correlate the baseline fractional anisotropy (FA) of the corticospinal tract in the damaged hemisphere with the initial motor performance as assessed by the FMA-UA and the changes in EEG-based neurofeedback training performance in VR (i.e., last training session versus first training session).

For the diffusion MRI preprocessing, we followed the HCP processing pipeline^3^. We then, modeled each voxel using a multi-compartment fiber orientation distribution (FOD) approach. Finally, we obtained the diffusion tensor FA values using FSL and completed the deterministic FOD fiber tracking using the Quantitative Imaging Toolkit (QIT). For each patient, we created two tracts: a damaged corticospinal tract (CST) and an undamaged CST from an ROI covering the posterior limb of the internal capsule in each hemisphere.

### Statistical Analyses

We note that this is a pilot study with a small sample size (*n* = 4). However, we performed statistical analyses at the group and individual subject levels to provide a general indication of the significance of any observed changes. We encourage the reader to interpret these statistical analyses with caution.

We assessed the normality of the distribution of all data using the Shapiro-Wilk (S-W) normality test. We used one-sample *t*-tests to determine whether there was a significant difference between the patient’s ERD values and the mean ERD values of a healthy population from prior literature (Pfurtscheller and Aranibar, 1979; Pfurtscheller et al., 2006). We performed paired *t*-tests to compare pre- and post-intervention means for mu and beta bands on the same continuous, dependent variable (ERD %), and for pre- and post-intervention clinical scales. We used repeated measures (RM) ANCOVAs (one RM-ANCOVA for each ROI as a seed region using “sessions” (pre and post-intervention sessions) as a factor and lesion size and lesion overlap on the CST as covariates) in the CONN toolbox in Matlab to compare combinations of pairs between the four ROIs (the following six FC pairs: left M1-right M1, left PMd-right PMd, left M1-left PMd, left M1-right PMd, right M1-left PMd, right M1-right PMd) and to compare the intra/inter-hemispheric connectivity across sessions. We used one RM-ANOVA to compare the recruitment curve across session using “thresholds” (RMT, 110, 120, 130, 140, 150%) and “sessions” (pre and post-intervention sessions).

We used non-parametric group comparisons using the Kruskal-Wallis *H*-test for the EMG data. Finally, we performed Pearson’s correlations between the strength of the CST in the damaged hemisphere (as measured by the FA value) and the lesion size/location with the initial motor performance (as measured by the FMA-UA score) and also with the changes in EEG-based neurofeedback training performance (last training session versus first training session).

For all statistical comparisons, the significance level was set to 5% (*p* < 0.05). All statistical analyses were done using IBM SPSS 20 (SPSS Inc., Chicago, IL, United States), R (The R Foundation for Statistical Computing Platform, version 3.5.2), and Matlab R2017a (The MathWorks, MA, United States).

## Results

### Simulator Sickness in VR Training

**FIGURE 4:**
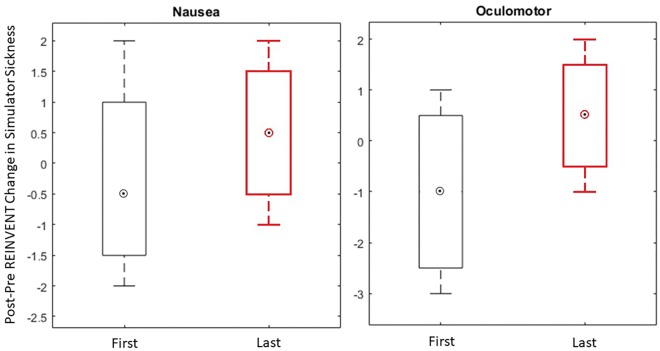
Change in simulator sickness following REINVENT use in the domains of Nausea and Oculomotor disorientation for the first and the last sessions of the BCI-VR intervention. A box and whisker plot showing medians and standard deviations are illustrated.

A key concern of using a VR-based BCI over multiple sessions is the possibility of discomfort resulting from VR-induced simulator sickness. Simulator sickness refers to symptoms similar to motion-induced sicknesses, such as dizziness and nausea, following visually-induced simulations (e.g., from head-mounted virtual reality) (Kennedy et al., 1993). We measured simulator sickness before and after the first and last session of the BCI-VR training. Across all patients, a paired-samples *t*-test revealed no significant differences before and after REINVENT use in either the first or last session for either the nausea or oculomotor subscales [first session: Nausea, *t* (3) = 0.2928, *p* = 0.79, Oculomotor, *t*(3) = 1.0954, *p* = 0.35; last session: Nausea, *t*(3) = −0.7746, *p* = 0.50, Oculomotor, *t*(3) = −0.7746, *p* = 0.50]. The average change in simulator sickness following REINVENT use was small for both nausea (*M* = 0.13, *SD* = 1.46) and oculomotor (*M* = −0.25*, SD* = 1.67) subscales (Figure 4). This suggests that repeated VR intervention could be feasible and does not induce increases in simulator sickness.

### Neurofeedback Performance

We extracted the neurofeedback performance in terms of training score in VR and ERD power between the first and the last session of the intervention block.

#### Training Score

**FIGURE 5:**
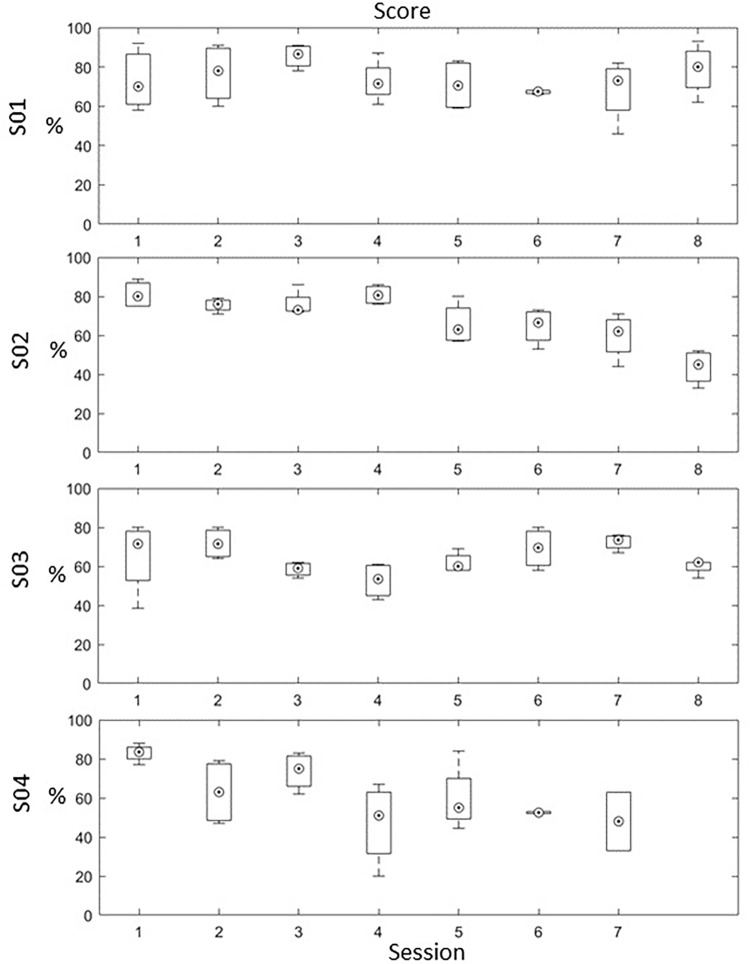
In-game training score for each patient across the 8 sessions (each session score is an average of the 4 training blocks). A box and whisker plot showing medians and standard deviations are illustrated.

In terms of training performance (measured as successful when the virtual hand reached the target), only S01 had an increased score of 7.3% between the first (*M* = 73.3, *SD* = 17.2) and the last session (*M* = 80.6, *SD* = 9.6) while the rest of the patients showed a decrease over time. Specifically, S02 had a decreased score of 37.1% between the first (*M* = 80.9, *SD* = 7) and the last session (*M* = 43.8, *SD* = 8.9); S03 had a decreased score of 5.4% between the first (*M* = 65.4, *SD* = 8.7), and the last sessions (*M* = 60, *SD* = 4) and S04 had a decreased score of 32.5% between the first (*M* = 80.5, *SD* = 5) and the last sessions (*M* = 48, *SD* = 21.2). A paired-samples t-test revealed a significant difference between the first and the last sessions, only for S02, *t*(3) = 6.671, *p* = 0.007 (Figure 5). However, by examining the session scores, it is clear that there was wide variability in training score individuals and across sessions. This suggests that the patient’s ability to control the neurofeedback was highly variable, and may depend on variables such as mood, motivation, attention, and fatigue.

#### ERD Power

**FIGURE 6:**
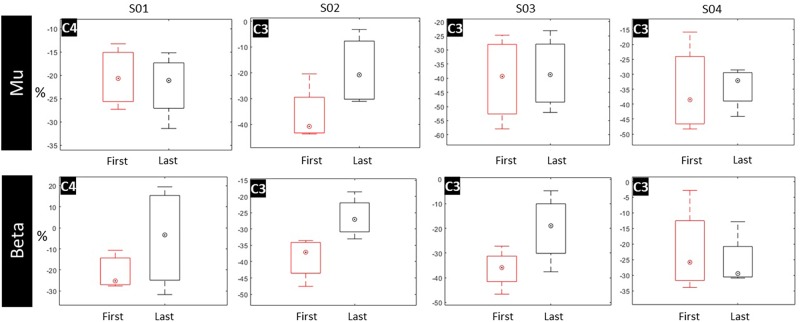
ERD values for mu and beta bands pre- and post-intervention for C3 and C4 electrodes. A box and whisker plot showing medians and standard deviations are illustrated.

Because both movement and imagery are associated with mu and beta rhythm desynchronization (McFarland et al., 2000), we anticipated a stronger ERD (in terms of higher negative percentage compared to baseline) at the end of the intervention. However, a paired-samples *t*-test revealed no significant differences between the first and last session across patients (Figure 6).

### *Post hoc* EEG Analyses

**FIGURE 7:**
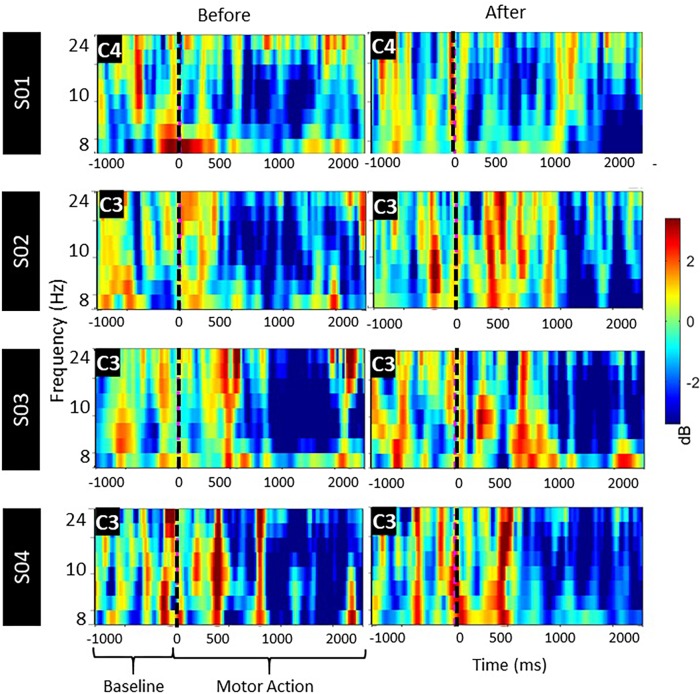
ERSP activation maps, pre and post stimulus between mu and beta bands over the lesioned side during motor attempt. Significant ERD is illustrated with blue.

We extracted the ERSP maps for qualitative purposes as a time/frequency representation of ERD/ERS for all pre- and post-intervention trials. Maps illustrate a clear desynchronization between 8 and 24 Hz compared to baseline for all patients (Figure 7).

#### ERD Comparisons With Healthy Population Data

We then compared the mean ERD for our patients with the mean for a representative healthy population, reported previously (Pfurtscheller and Aranibar, 1979; Pfurtscheller et al., 2006). Because the healthy population data only reported the frequency range of the mu band, we compared these data with the mu ERD from the patients in our study. In this way, we were able to quantify the difference between the evoked ERD of stroke patients compared to healthy individuals.

**FIGURE 8:**
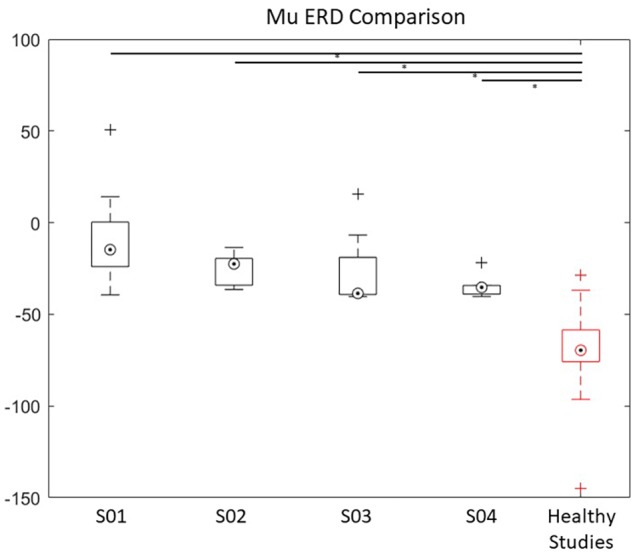
ERD power of the Mu band over the lesioned side of the four stroke patients compared with healthy population data (Pfurtscheller and Aranibar, 1979; Pfurtscheller et al., 2006). ^∗^ indicates significance of *p* < 0.05. A box and whisker plot showing medians and standard deviations are illustrated.

We performed an independent one-sample *t*-test for each patient, and it revealed significant differences between all patient ERDs compared to the mean ERD of the healthy population (Figure 8). Specifically, for S01, ERD (*M* = −10.8, *SD* = 21.3) was lower than the mean healthy ERD value of −74.5, with a statistically significant mean difference of 63.6, 95% CI [51.95–75.3], *t*(15) = 11.591, *p* < 0.05. For S02, ERD (*M* = −25.3, *SD* = 8) was lower than the mean healthy ERD value of −74.5, a statistically significant mean difference of 49.2, 95% CI [42.1–56.3], *t*(7) = 16.367, *p* < 0.05. For S03, ERD (*M* = −27.3, *SD* = 19.3) was lower than the mean healthy ERD value of −74.5, a statistically significant mean difference of 47.2, 95% CI [30–64.5], *t*(7) = 6.479, *p* < 0.05. Finally, for S04, ERD (*M* = −34.8, *SD* = 5.8) was lower than the mean healthy ERD value of −74.5, a statistically significant mean difference of 39.7, 95% CI [33.9–45.5], *t*(6) = 16.789, *p* < 0.05 (Figure 8).

### Correlation Analyses

Resting-state EEG alpha oscillation synchrony was previously determined to be related to cognitive and motor performance in patients (Dubovik et al., 2012, 2013). Analysis of the interhemispheric asymmetries might provide a valuable neurophysiological parameter to determine prognosis and follow-up of patients (Cicinelli et al., 2003). In addition, baseline motor impairment level may impact the ability to control the BCI feedback. We, therefore, assessed the relationship between VR task (score) and the resting-state alpha, alpha hemispheric asymmetry or motor impairment score (FMA). Specifically, a strong significant negative correlation was observed between the VR task score and the FMA score (*r* = −0.96, *p* = 0.04) but not for resting-state alpha (*r* = −0.83, *p* = 0.17), nor the hemispheric asymmetry of alpha (*r* = −0.82, *p* = 0.18). This suggests that baseline motor impairment impacts a person’s ability to control the BCI, similar to previous findings (Liew et al., 2016). In addition, the results for the EEG correlations showed a strong relationship, but this is not significant, likely due to the small sample size and high variability.

### *Post hoc* EMG Analyses

Because we designed the REINVENT system to match patients’ motor ability with the best available interfacing technology, we also performed an offline analysis of muscle responses in all patients to validate REIVENT’s ability to measure training benefits across modalities and to examine the potential of using EMG data as an interfacing modality for those with a degree of active movement.

**FIGURE 9:**
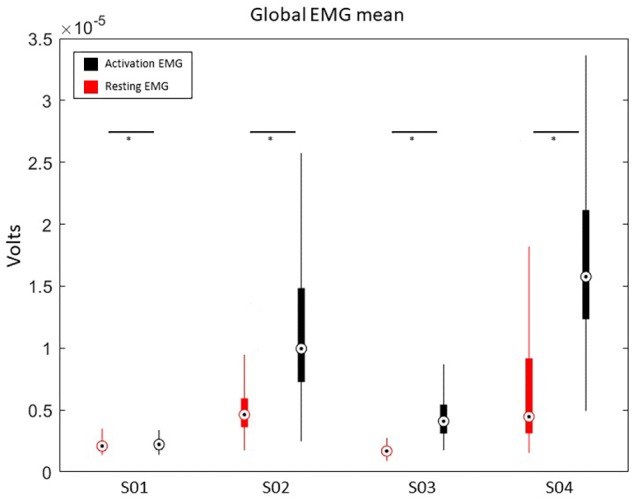
Average EMG activation during baseline (resting) and motor action (muscle activation). ^∗^ indicates the significance of *p* < 0.05. A box and whisker plot showing medians and standard deviations are illustrated.

After analyzing the EMG data from all baseline sessions (*N* = 640) and trials during BCI training, we extracted the mean EMG (in mV) from all subjects. This allowed us to quantify muscle activation differences between rest and during motor intention. To compare the mean EMG activation between patients, we used a Kruskal-Wallis *H*-test, which revealed significant differences in signal amplitude χ^2^(3) = 2044.43, *p* < 0.001 as well as in resting-state amplitude χ^2^(3) = 1711.63, *p* < 0.001. Current data found EMG differences between patients for both resting-state and muscle activation and also differences within each patient for EMG signal during activation versus rest periods (Figure 9). This suggests that active EMG signals can be distinguished from baseline rest in all of our patients, across different motor impairment levels.

**FIGURE 10:**
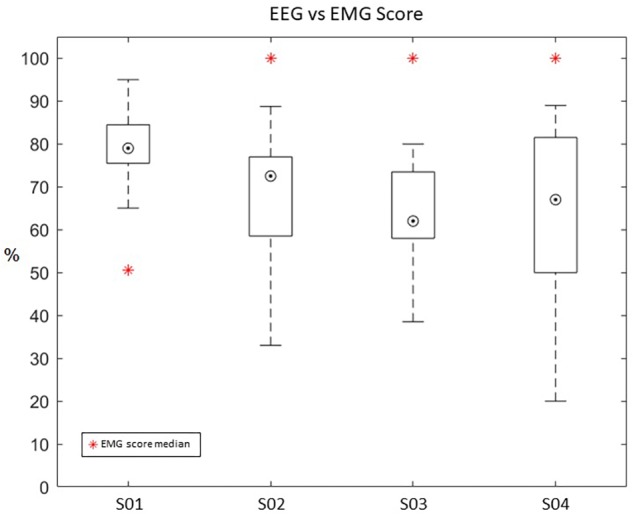
Re-calculated average score from EMG data (shown as a blue and red circle) compared to the average EEG-based score (shown as a box and whisker plot) during training. A box and whisker plot showing medians and standard deviations are illustrated.

We, then, examined whether EMG neurofeedback might have been more successful for patients than using EEG neurofeedback. In particular, we found that patients with less motor impairment showed reduced performance in VR training when using EEG neurofeedback. Therefore, we re-calculated the training score for all patients using the EMG data and compared it with the performance levels calculated using EEG (Figure 10).

Patients with higher motor ability showed a higher success rate when using EMG neurofeedback than when using EEG neurofeedback. Specifically, S02 (EEG *Mdn* = 72.5, EMG *Mdn* = 100), S03 (EEG *Mdn* = 62, EMG *Mdn* = 100), and S04 (EEG *Mdn* = 67, EMG *Mdn* = 100) had better results using EMG neurofeedback. Only patient S01 (EEG *Mdn* = 79, EMG *Mdn* = 55), who had a lower motor ability and muscle flaccidity, showed better performance using EEG neurofeedback. This suggests that motor impairment level should be used to determine the modality of neurofeedback given to patients.

### MRI, TMS, Behavior

#### TMS Sessions

Patients participated in two TMS sessions – one pre-intervention and one post-intervention – to assess the functional integrity of the corticospinal tract, or brain-to-muscle pathway, in patients (Stinear and Byblow, 2017). This measure has been related to post-stroke motor recovery and ability to benefit from therapy.

**FIGURE 11:**
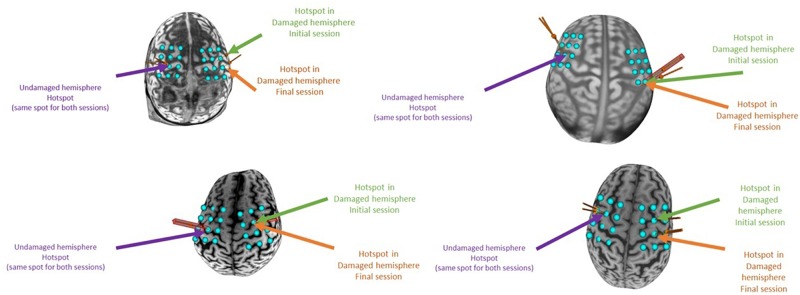
Individual motor hotspot locations in each session. On each individual brain, a blue acquisition grid is displayed around the sensorimotor cortex. Using this grid, we localized the motor hotspot during the initial and final session. The site where the hotspot was localized for each session is displayed with a colored arrow in both the damaged and undamaged hemispheres. In patients S01 and S04, the motor hotspot in the damaged hemisphere was found at a different grid node between the two sessions.

In the four patients (Table 2), we localized the motor hotspot in the undamaged hemisphere around the M1 area (i.e., the hand knob). This localization was reliable between pre- and post-intervention assessments (Figure 11). At this location, we acquired the RMT at the same intensity between the two sessions (Pre: 51.3 ± 6.4, Post: 52.3 ± 5.1). It was difficult to localize the motor hotspot in the damaged hemisphere; therefore, we had to use a higher stimulator intensity to evoke an MEP. At this location, only one patient (S04) had a reachable RMT during the first session (71%). For the other patients, as RMT was not reachable at each session, we determined the active motor threshold (AMT) in both pre and post-intervention sessions. Interestingly, however, while patient S01 did not have a reachable RMT pre-intervention, he had a reachable RMT post-intervention (which was maintained after his extended 16-session protocol, Vourvopoulos et al., 2019). In this patient, the motor hotspot localization from the AMT moved posteriorly to the parietal cortex for the RMT (in the post-intervention session).

**Table 2:** TMS motor hotspot.

	Undamaged hemisphere		Damaged hemisphere			
	RMT pre-int	RMT post-int	RMT pre-int	AMT pre-int	RMT post-int	AMT post-int
S01	48%	48%	n/a	68%	73%	68%
S02	58%	58%	n/a	75%	n/a	75%
S03	55%	55%	n/a	75%	n/a	75%
S04	44%	48%	71%	Not acquired	75%	Not acquired
Mean	51.25%	52.25%	Cannot be calculated due to missing data	72.7%	74%	72.7%
SD	6.40%	5.06%		4.04%	1.41%	4.04%

*RMT, Resting Motor Threshold; AMT, Active Motor Threshold; Pre-Int, Post-Int, Pre- and Post-Intervention. Results displayed are a percentage of stimulator output.*

We were able to reliably acquire the recruitment curves only in the undamaged hemisphere for all patients. Using a RM-ANOVA, we compared the recruitment curves across the two sessions and observed only an effect of the “thresholds” (meaning an increase of the MEP amplitude with increased threshold) but there was no effect of “sessions” nor an interaction effect (RM ANOVA: interaction “thresholds”x”sessions”: *F*(5) = 0.084, *p* = 0.99; thresholds: *F*(5) = 3.96, *p* = 0.006; sessions: *F*(1) = 0.275, *p* = 0.60).

#### fMRI ROI-to-ROI Analysis

We next examined changes in connectivity for each motor ROI-to-ROI pair using an ANOVA with time as a factor. We did not observe any significant differences in the ROI-to-ROI functional connectivity measurement between pre- and post-intervention at the group level as assessed using an integrated ANOVA in the CONN toolbox. Finally, we averaged the functional connectivity measurements for the intra- and inter-hemispheric motor network connectivity and observed also no significant changes between pre- and post-intervention in the intra-hemispheric connectivity in the damaged hemisphere. We also did not observe any statistically signitficant impacts of the lesion size or the lesion overlap with the CST on: (1) connectivity [mean connectivity session pre: 0.27 ± 0.36; mean connectivity session post: 0.19 ± 0.18; ANCOVA: time: *F*(3) = 0.92, *p* = 0.44, lesion size: *F*(1) = 0.82, *p* = 0.53, lesion overlap with the CST: *F*(1) = 8.49, *p* = 0.22], (2) the intra-hemispheric connectivity in the undamaged hemisphere [mean connectivity session pre: −0.10 ± 0.19; mean connectivity session post: 0.05 ± 0.19; ANCOVA: time: *F*(3) = 5.19, *p* = 0.11, lesion size: *F*(1) = 0.22, *p* = 0.72, lesion overlap with the CST: *F*(1) = 10.65, *p* = 0.19], or (3) the inter-hemispheric connectivity in the damaged hemisphere [mean connectivity session pre: 0.21 ± 0.19; mean connectivity session post: 0.29 ± 0.22; ANCOVA: time: *F*(3) = 0.74, *p* = 0.45, lesion size: *F*(1) = 0.13, *p* = 0.77, lesion overlap with the CST: *F*(1) = 3.12, *p* = 0.33].

#### Diffusion MRI

**FIGURE 12:**
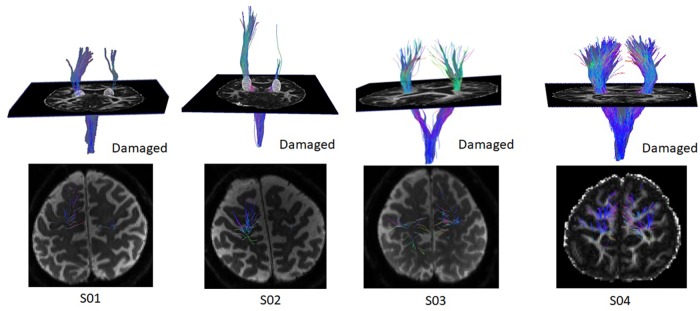
Individual corticospinal tracts in both hemispheres. The damaged hemisphere is represented on the right hemisphere in all images.

We obtained the CST tract in the damaged and undamaged hemisphere of each of the four patients. As shown in Figure 12, patients S03 and S04 overall had intact CST tracts in both hemispheres, whereas patients S01 and S02 had impaired CST tracts in the damaged hemisphere. We explored if the strength of the CST in the damaged hemisphere could predict the changes in EEG-based neurofeedback training performance. This analysis revealed no correlation (Pearson: *r* = −0.03, *p* = 0.96) between these measurements. We also performed a Pearson correlation analysis between the FA of the CST in the damaged hemisphere and the initial motor ability of the patients and observed a positive non-statistically significant trend with the initial FMA-UA score (*r* = 0.92, *p* = 0.07), suggesting a loose relationship between patients with a stronger CST in the damaged hemisphere and better initial motor ability.

#### Lesion Size and Lesion Location

We used the size of the lesion and the lesion overlap with the CST to explore the impact of the lesion location and size on the initial motor impairment (initial FMA-UE). There was no relationship between either the size of the lesion and the initial FMA-UE (Pearson: *r* = −0.81, *p* = 0.19), or the lesion overlap on the CST and the initial FMA-UE (Pearson: *r* = −0.47, *p* = 0.53).

We also explored if the lesion size and location had any impact on the EEG-based neurofeedback training performance. We found a strong positive, but non-significant, correlation between the changes in EEG-based neurofeedback performance and both the lesion size (Pearson: *r* = 0.84, *p* = 0.16) and the lesion overlap with the CST (*r* = 0.91, *p* = 0.09). Although these were not significant, these show a potential trend that patients with larger lesions and greater lesion overlap with the CST may have greater improvements on the EEG-based neurofeedback training performance.

#### Clinical Outcomes

Last but not least, in terms of clinical scales, we compared whether there was a difference in FMA-UE, MAS, or SIS before and after the 8 week intervention block, expecting an improvement in clinical scales. A paired-samples *t*-test yielded no significant group differences between pre- and post-intervention for all scales.

Nonetheless, patients S01, S03, S04 had increased FMA-UE scores, with S03 demonstrating a six-point increase in the FMA-UE. According to Page et al. (2012) this degree of increase in the FMA-UE meets the minimal clinically important difference threshold of 4.25–7.25 points. Finally, the self-reported SIS scores increased in all patients except one (S04), while the MAS remained stable (Table 3).

**Table 3:** Clinical scales pre- and post-intervention.

	FMA-UA		MAS		SIS	
Patient	Pre	Post	Pre	Post	Pre	Post
S01	13	14	2	2	45	75
S02	28	25	0	0	35	50
S03	37	43	1	1	10	60
S04	49	50	0	0	40	30

*FMA-UA, Fugl-Meyer Assessment – Upper Extremity; MAS, Modified Ashworth Scale; SIS, Stroke Impact Scale.*

## Discussion

In this pilot study, we described the use of an EEG-based BCI for motor rehabilitation that provides biologically-relevant neurofeedback in head-mounted virtual reality (REINVENT) with four chronic stroke patients across different motor impairment levels. We demonstrated that VR-based neurofeedback may be feasible for stroke patients with motor impairments for prolonged, multi-session use. We also suggest that the source of neurofeedback should be tailored to the individual’s impairment level and that for individuals with more motor ability, EMG-based feedback may be more useful. Overall, the current results contribute toward our understanding of how BCI-VR training can be used for chronic stroke patients with different levels of motor impairment.

First, we found that repeated use of a BCI with head-mounted VR-based neurofeedback (8 sessions of 1.5 h each) was feasible and tolerable for stroke patients across a variety of motor impairment levels. The overall set-up process took about 20 min per individual, including setting up the 8-channel EEG and 4-channel EMG systems and putting on the HMD-VR over the EEG cap. However, there were no issues with simulator sickness following any of the sessions and no other complaints about pain, discomfort or fatigue. Overall, patients were enthusiastic about using REINVENT and appreciated the new form of therapy. Despite variable motor results after using REINVENT, patients self-reported positive changes in overall quality of life on a comments form and asked to continue using REINVENT after the study was completed. This suggests that the use of multiple sessions of a VR-based BCI paradigm not only could be feasible but potentially enjoyable for individuals after stroke.

Second, we found that the EEG-BCI seemed to have the greatest positive effects for the patient with the worst motor impairments (patient S01), and little-to-no effects for individuals with more motor ability (e.g., more volitional movement of the affected limb). Patient S01 was the only one to have significant changes in cortical physiology, as measured by TMS as the appearance of a motor-evoked potential during rest, and he also had large improvements in SIS following REINVENT use. He was also the most successful at controlling the EEG-BCI feedback and showed improved performance following REINVENT training. In contrast, the other three patients all had volitional movement of the affected hand and wrist and did not show improved performance with REINVENT training. They also showed many fewer and more variable changes in behavior and neural assessments. This finding mirrors previous work showing that individuals with greater motor impairments after stroke show the greatest benefits from real-time fMRI neurofeedback (Liew et al., 2016). One potential hypothesis to explain this finding is that in individuals with worse motor impairment, there are fewer inputs to and outputs from the damaged motor cortex, hence poorer motor ability. Given this, these brain areas may be more flexible to neuromodulation and may be more easily trained because these regions are not being actively engaged for other tasks. On the other hand, in individuals with volitional movement, these brain regions may already be actively recruited through more naturalistic processes (e.g., trying to move one’s arm on a regular basis) and may be less flexible to learn new patterns imposed by the neurofeedback training.

On the other hand, individuals with volitional movement may show greater benefits from a form of neurofeedback that strengthens their existing brain-to-muscle pathways. Our *post hoc* EMG analysis showed that if individuals with volitional movement had been given EMG-based feedback, instead of EEG-based feedback, they would have shown much better BCI control and performance. This was the opposite for Patient S01, who would have performed worse with EMG-based feedback. Although more research is needed in a larger and more diverse sample, these data provide some insight into how BCIs could be personalized to each patient’s needs. For those without volitional motor control, learning to control the damaged hemisphere at all, using broad motor frequency bands was successful. However, for those with some motor control or at least muscle activity, harnessing the individual’s own already established pathways may be more effective. This could potentially be done with EEG, by matching the neurofeedback to the specific ipsilesional brain activity evoked when the individual moves, or with EMG, which by nature utilizes an established pathway from the brain to the muscle. A flexible BCI that collects multimodal data (e.g., EEG and EMG), and can provide a tailored neurofeedback signal, may have the best potential for improving recovery.

Overall, these results show that the training paradigm was feasible and safe. However, there were no significant group changes in clinical scales or in brain imaging metrics following REINVENT. This is perhaps not surprising, given that three of the individuals with greater motor ability did not show improved performance on the BCI, and thus would be unlikely to show improvements in motor function or brain activity. The one consistent improvement was in the SIS, which improved in three of the four patients, and could be more related to the social interactions of engaging in a novel therapy and therapy team. In terms of actual motor changes, the most notable change was the detection of a resting motor threshold in patient S01 after neurofeedback training, and he was also the only person to improve on the neurofeedback training. We hypothesize that for the other three patients, using EMG-based neurofeedback will have more positive effects. It is possible that 8 sessions might be too few sessions to show marked changes in either behavior or intrinsic brain activity; future studies should explore this with longer study duration. It is also worth noting that there is high variability in BCI performance across sessions, suggesting that individual state factors, such as fatigue, motivation, and attention may have a strong influence on patients’ ability to control the BCI. Patients also reported experimenting with trying out different strategies to control the BCI across days, which could also have introduced variability in overall BCI performance and resulting behavioral and neural changes.

## Limitations

Although this study collected and explored 160 EEG datasets (128 motor related and 32 resting-state data) along with pre- and post-intervention MRI, TMS, and clinical behavior datasets from stroke patients, it was limited by its sample size (*N* = 4). Our findings, therefore, are preliminary, have limited statistical power, and should be interpreted with caution. In addition, the statistical outcomes relating to our measurement (efficacy of the proposed technique) and the comparisons presented here are exploratory and not confirmative. Furthermore, increasing the number of sessions per patient could also have resulted in more positive results. Finally, as noted above, given the wide range of motor impairment levels across our four patients, there was significant variability in results. Future studies should focus on testing EEG-based BCIs with VR in a wider population of individuals with severe motor impairments.

## Conclusion

Overall, in this study, we illustrated a novel architecture with multimodal interfaces for widening the inclusion criteria into VR rehabilitation and training. We showed the effect of an EEG-based VR BCI in stroke survivors with a wide range of motor disabilities and identify a potential clinical profile of those who can benefit from an EEG-based interface. This pilot data suggests that patients with more severe motor impairments achieve the maximum benefits of a BCI paradigm, while those with active movement may benefit more from EMG feedback in a multimodal platform. Finally, this VR-based platform is feasible for use by individuals with stroke across repeated sessions, opening the potential for new VR-BCI paradigms for stroke rehabilitation.

## Data Availability

The datasets generated for this study are available on request to the corresponding author.

## Ethics Statement

The experimental protocol was approved by the University of Southern California Health Sciences Campus Institutional Review Board (IRB), and written informed consent was obtained from all participants upon recruitment in accordance with the 1964 Declaration of Helsinki.

## Author Contributions

AV participated in the development of the VR and neurofeedback software and collected and analyzed the EEG data. AV and OP designed the technical specifications underlying the experiment. OP collected and analyzed the EMG data. SL collected and analyzed the TMS and MRI data. MN collected all the behavioral data and performed the clinical assessments pre- and post-intervention. DS and EJ recruited the patients. AV, OP, SL, S-LL, and MN interpreted the data. S-LL designed and supervised the study. All authors revised and approved the current version of the manuscript and defined and designed the research study.

## Conflict of Interest Statement

The authors declare that the research was conducted in the absence of any commercial or financial relationships that could be construed as a potential conflict of interest.

## Footnotes

1 http://protocols.humanconnectome.org/CCF/

2 http://www.nitrc.org/projects/conn

3 https://github.com/Washington-University/HCPpipelines/tree/master/DiffusionPreprocessing
